# Comparison of the efficacy of COVID-19 responses in South Korea and the United States

**DOI:** 10.1080/16549716.2024.2370611

**Published:** 2024-08-13

**Authors:** Oliver Choi, Sunjoo Kim

**Affiliations:** a The Fessenden School, West Newton, Massachusetts, USA; b Department of Laboratory Medicine, Gyeongsang National University Changwon Hospital, Changwon, Republic of Korea; c Department of Laboratory Medicine, Gyeongsang National University College of Medicine, Jinju, Republic of Korea; d Institute of Medical Sciences, Gyeongsang National University, Jinju, Republic of Korea

**Keywords:** COVID-19, SARS-CoV-2, response, contact tracing, vaccine

## Abstract

**Background:**

The COVID-19 pandemic devastated many countries worldwide by causing large numbers of fatalities. In our research, we wanted to answer the question: Why was there such a large difference in the mortality rate between South Korea and the United States? This is because many East Asian countries, such as Korea, had a lower mortality rate than many countries, including developed ones, across the world – the mortality rate of South Korea was about five times lower than the United States.

**Methods:**

This study comprehensively compares strategies used to address the COVID-19 pandemic in two different countries: South Korea and the United States. The various aspects of these two countries’ responses are examined, including initial response, information dissemination and public compliance, mitigation strategies, and vaccine rollout and their impacts.

**Results:**

Early and widespread testing, rigorous contact tracing, the clear release of government information, and an organized vaccine rollout powered a proactive approach in South Korea. The United States had a contrasting response consisting of delayed and more decentralized measures, where testing lagged due to varying policies and the political controversies facing vaccine distribution.

**Conclusions:**

We signify the gravity of rapid response and testing, clear communication, and efficient vaccine distribution, as we believe this could correlate with a lower mortality rate. In addition, we discuss future directions, including the need for a specific health infrastructure and protocol against highly infectious outbreaks.

## Paper context

- **Main findings:** The study suggests strategies that may be effective ways to reduce fatalities during a pandemic through a comparative analysis of COVID-19 responses in South Korea and the United States.
- **Added knowledge:** This review further consolidates the importance of an effective defense strategy involving testing, contact tracing, information dissemination, and vaccine rollouts.
- **Global health impact for policy and action:** There is a need for the development of a specific pandemic response infrastructure against fast-spreading and fatal viruses involving effective policies that take into account both the freedom and health of citizens.

## Introduction

The world-altering COVID-19 pandemic emphasized the importance of having well-organized and efficient public health responses for infectious diseases. In 2020, the pandemic spread worldwide, affecting most, if not all, countries. South Korea, from here on referred to as Korea, was specifically examined because while much research has been done regarding the differences in COVID-19 mitigation strategies between China or Japan and the United States [1,2], there have not been many studies specifically highlighting Korea’s unique strategies that could have impacted its successful outcome in reducing fatalities. The objective of this paper is to answer the question: *Why was there such a large difference in the mortality rate between South Korea and the United States*? We aimed to discover why the mortality rates in these two developed nations with similar socioeconomic and political characteristics and robust medical infrastructures varied so much; Korea possessed a mortality rate about five times lower than the United States [3].

This report examines the two different countries’ mitigation strategies for preventing the spread of COVID-19, recognizing areas to highlight and learn lessons from considering another decreases-led pandemic. This could help prompt future health policies since we provide a contrasting view of policies Korea and the United States implemented.

On January 20, 2020, the first confirmed case of COVID-19 was reported in Korea [4]. The MERS-CoV (Middle East Respiratory Syndrome) outbreak in 2015, caused by a different strain of coronavirus, resulted in 39 deaths (with fatality at 20.4%), raising social unrest and demand for stricter measures to prevent the spread and resultant deaths from viruses. Owing to its experience with MERS, Korea created a specialized framework focusing on identifying and treating patients before COVID-19 spread further [5].

The United States confirmed its first case of COVID-19 on January 20, 2020, the same day as Korea [6]. The United States had not experienced a recent widespread virus-led outbreak like MERS; therefore, it had not developed a systemic and effective protocol. Resultant inadequate testing contributed to a slow initial response, and further delays in tracing and isolating cases were also common. There was a lack of a unified response since the United States depended on state and local health departments, while the federal government did not mandate lockdowns in possible COVID-19-infected areas, leading to confusion among citizens and troubled healthcare systems [7].

Both Korea and the United States possessed strategies to manage pandemics before COVID-19 took place; learning from the MERS outbreak, Korea amended the Infectious Disease Control and Prevention Act, making it easier for the government to acquire regulations to combat the COVID-19 outbreak [8]. By contrast, the United States had to overhaul its pandemic response, which was mainly designed for influenza-like viruses with low fatality rates, to reflect a new and unknown coronavirus that has a fast-spreading nature and higher rates of fatality and mortality.

This research found that the most effective way to reduce fatalities in a pandemic is to create specific public health measures accustomed to the virus in terms of its strength and its features. Governments, using these measures, must communicate with the public while combating misinformation, implement response and testing frameworks, and work towards distributing vaccines, especially for high-risk populations, so that it prevents fatalities.

## Methods

To collect data for our research, we utilized health reports from health organizations across the world, such as the World Health Organization (WHO), the U.S. Food and Drug Administration (FDA), and the Center for Disease Control and Prevention (CDC). As supplementary sources, we used disclosed governmental information and data. We researched and cited published articles from Google Scholar and PubMed to investigate the pandemic’s diverse consequences across East Asia, more specifically Korea, and the United States.

In this paper, the mortality rate of a country is defined by the number of fatalities over the population count, while as the fatality rate is defined by the number of fatalities over the number of cases.

The different types of responses these two countries enacted during COVID-19 was separated into four categories: initial response, information dissemination and public compliance, mitigation procedures, and vaccination strategies, alongside their impact. These indicators were chosen because they showed the most variation in the ability of the two countries to respond to the pandemic.

Initial response was included as a factor because research showed that a more effective initial response to a pandemic could alter its outcome [9]. The similarity between countries that generally had a better response to COVID-19 and a lower resultant mortality rate is that they all implemented testing, contact tracing, travel restrictions, and lockdown measures in the early stages. For example, considering the East Asian nations that met our criteria, which considered East Asian countries with a population of over 50 million, China and South Korea ramped up their testing procedures in late January and Japan in early February [10–12]. This was very early compared to the United States – testing became more widespread through drive-through testing clinics and commercial lab testing in the United States during early March [13].

Information dissemination and public compliance were included as a factor because there were vast differences in how information was disseminated among many countries and how the public complied. Many countries, including the United States, had trouble with false information being spread regarding COVID-19 and vaccines. This sparked a lower compliance rate in terms of vaccination and mask usage, causing the virus to spread at an accelerated rate and the mortality rate to spike. It is crucial to highlight different fact-check news programs and special broadcasting procedures Korea implemented using daily briefings to combat misinformation and disseminate important information regarding recent outbreaks.

Mitigation procedures were included as a factor because preventing COVID-19 cases blocks resulting deaths, as COVID-19 causes many consequences, such as pneumonia, which could be fatal. As Korea and the United States are both democracies, the governments of both countries worked towards balancing the freedom of their citizens and the restrictions in place due to COVID-19. While Korea implemented a unique dynamic four-tier social distancing mechanism, which had different restrictions based on the average weekly spread of COVID-19, the United States’ restriction strategies were politicized, causing societal problems and a sudden spike in COVID-19 in some areas. This indicator was especially important since it sets a future path for the development of public health infrastructures and guidelines inspired by Korea’s strategy.

Vaccination strategies and impact were included as indicators because vaccines were the most crucial tool in terms of preventing fatalities among people infected with COVID-19. Since our area of focus was discovering different strategies South Korea and the United States took to lower the mortality rate, we figured that these two countries could have implemented different strategies to vaccinate their respective people. We found that South Korea had a higher percentage of fully vaccinated people compared to the United States, which could have contributed to a lower mortality rate in South Korea. In addition, all the East Asian countries that met our specific criteria, as explained above, had a higher percentage of fully vaccinated people compared to the United States, proving that there could be a correlation between the percentage of people fully vaccinated and the average mortality rate in a nation. It is crucial to discover specific vaccination strategies South Korea used to successfully achieve such a high percentage of fully vaccinated people and a low mortality rate, as it could benefit other nations to implement these strategies for future infectious crises.

We also focused on societal and economic problems, a hidden issue behind cases and mortalities, when evaluating outcomes.

Data analysis was performed by comparing data and performing computations with our visualizations via Google Sheets and *Our World in Data*: a free, open-source program.

## Comparisons of initial response

We analyzed the public health measures implemented in the early period of the COVID-19 pandemic to uncover the reasoning behind different pandemic outcomes in Korea and the United States. It is evident that to control a viral outbreak, rapidly identifying and isolating cases through extensive testing and contact tracing is needed to prevent further cases and mortalities. The experiences of Korea and the United States demonstrate how these strategies can substantially affect a nation’s pandemic management trajectory.

After gaining valuable insights from the 2015 MERS outbreak, Korea promptly activated a sophisticated response system. They built up negative pressure rooms to isolate virus-infected patients at the emergency department for every tertiary care hospital and supplied medical equipment or protective gears to these hospitals. They also educated and trained medical personnel with scenarios of highly infectious disease outbreaks. They set up an emergency-use authorization (EUA) system for in vitro diagnostic tests. They also switched this policy from the national central laboratory test to regional laboratories to reduce the turnaround time. The law on personal information protection changed, making an exception for the sake of public health during the infectious disease crises. Shortly after identifying its first case, the nation dramatically increased its testing capacity – before the end of May, the daily testing capacity rose to 34,193 tests [14]. Many individuals could be tested rapidly using either public health centers or regional tertiary care hospitals [15]. By the time Korea reported 7,382 confirmed cases on March 9, 2020, it had conducted a total of 195,266 tests – an early and aggressive testing strategy that played a crucial role in preventing the spread of the virus [16].

Concurrently, a comprehensive contact tracing operation was launched. Authorities tracked the mobility of confirmed cases using various data, including credit card transactions and GPS data from mobile phones [17]. The government immediately notified those at risk of exposure using cell phones, notifying people of the need to quarantine. This strategy and the public dissemination of information regarding where particular patients had traveled helped Korea restrict the movement of people and, therefore, the spread of coronavirus.

By contrast, the United States’ approach to testing and contact tracing at the onset of the pandemic was significantly problematic. Prior to reporting a comparable number of confirmed cases (7,283) on March 11, 2020, the country had only conducted 19,214 tests, approximately ten times fewer than Korea [16]. Furthermore, from April 15 to June 25, 2020, Korea maintained a relatively high test-to-confirmed-case ratio of over 200. By contrast, the United States had a much lower ratio, barely reaching 17.5, indicating that Korea took more extensive precautionary measures to identify cases [16].

The United States, overall, had a lenient approach in terms of activating widespread testing and contact tracing, causing it to become inconsistent between states and communities. Efforts to expand this system were taken only after the pandemic was prevalent and peaking across the country. With less restrictive measures taken by some states which condemned lockdown and presented challenges regarding logistics, the government encountered problems while identifying and isolating infectious people. To prevent a virus from becoming a pandemic, if possible, the government should work toward controlling its spread at the start of the outbreak to mobilize resources given the provided time.

## Information dissemination and public compliance

Information regarding virus response protocols, like at-home measures according to the location of recent outbreaks, that are shared swiftly with clear transparency is known to ensure public awareness and compliance. We evaluated the impact that different approaches in Korea and the United States had on information dissemination and public compliance.

In the early stages of the outbreak, the Korean government provided its citizens with accurate information as quickly as possible. Through effective communication strategies like daily briefings and real-time alerts, informing the public about the severity of the situation and necessary precautions gained public compliance; this is necessary when limiting social conflict. By disclosing the locations visited by confirmed cases, the government encouraged possibly infectious candidates to undertake a test.

Despite Korea’s success at reducing both cases and fatalities during the early period of the pandemic by locating and transparently sharing details regarding patients, it faced criticism over possible privacy violations. The Korean government collected delicate personal details regarding COVID-19 patients, including but not limited to their credit card and phone usage, address, age, profession, sex, and name [18].

During the start of the pandemic, the United States government disposed of conflicting messages from state to state and community to community regarding the severity of the virus and appropriate strategies to prevent further spread. Due to the diverse message citizens received, the United States government faced challenges when providing citizens with a set of accurate and straightforward information, unlike Korea, where the federal government agency quickly set regulations that were sent out with various media sources to reach public attention. The United States’ lack of consistent communication resulted in different public responses, with varying levels of state and community compliance [7,19]. Despite the CDC’s recommendation to use masks and prevent viral transmission, many Americans chose not to wear them, making virus control more difficult. Conflicting political beliefs with public health recommendations, including mask-wearing, played a major role in decreasing compliance.

Most Koreans complied with donning N95 masks and assisted with efforts to trace down the COVID-19 spread. According to a poll directed by Gallup, approximately 90% of Koreans stated that they wear masks when going out [20]. This percentage is considerably higher compared to other nations worldwide, including the United States – the number of adults in the United States claiming they wear masks in public reached 70% in June, nearly five months after the first COVID-19 case, according to a YouGov poll. The compliance rate remained between 75% to 80% afterward until 2021, nearly 10% to 15% lower than Korea [21].

The use of masks could have differed vastly because of the different measures the governments put in place to encourage citizens to utilize masks. Through various commercials created by the government, Koreans were educated regarding the effectiveness of wearing masks and instructions on how to wear them. These advertisements were made to target specific audiences, with some broadcasted through channels directed at children and others in major news channels [22]. In addition, in Korea, if people did not comply with wearing masks, the government would fine 100,000 KRW, which is about $75. This law was mandated not only indoors but also outdoors in any public space. In addition, people had to wear ‘medically-approved’ masks, which were masks the government approved to be effective in preventing the spread of COVID-19, such as KF-94’s. However, Korea did face a mask shortage; since mask production in Korea was limited, when the demand for masks escalated, the government intervened in mask distribution to ration supplies to ensure everyone could protect themselves; citizens could get two masks per person per week at maximum [23].

**Figure 1.**
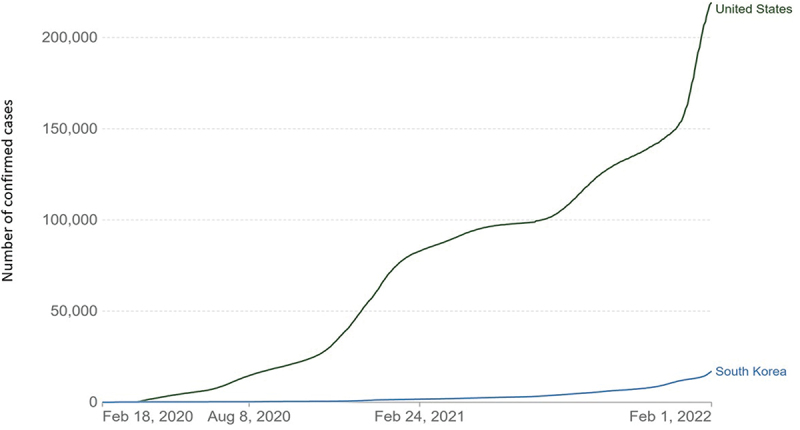
Cumulative confirmed COVID-19 cases in the early period of 2020 to early 2022 (reproduced with permission from OurWorldInData.Org).

In contrast, the United States had different policies regarding mask usage, which varied by state; although the CDC recommended everyone utilize masks, some states did not implement measures to enforce mask usage in public. The CDC did mention that ‘loosely woven cloth products provide the least protection’ and that ‘well-fitting disposable surgical masks and KN95s offer even more protection;’ however, there were no regulations that specified a specific type of mask citizens had to wear when there was a mask mandate [24]. This could have contributed to a faster spread of COVID-19 in the United States during the early stages of the pandemic, as shown in this figure (Figure 1).

Learning from the experiences of Korea and the United States, we can understand that using clear and consistent communication acquires public compliance during a health crisis, helping the nation take more control and, thereby, restricting the spread of the virus. In addition, the disparity shown by these two countries explains how public trust and willingness to learn and comply with regulations can be crucial factors in pandemic outcomes.

## Mitigation procedures

Mitigation measures are techniques that regulatory bodies use to control the spread and effects of a disease’s outbreak. To mitigate the spread and mortalities caused by COVID-19, Korea implemented a dynamic four-tier social distancing mechanism; the government altered the level of social restriction by considering infection and mortality rates to maintain an acceptable threshold. The levels ranged from Level 1, which enforced basic limitations, such as preventing large gatherings and maintaining a safe distance, to Level 4, where stringent measures, including banned social dining with two people and restricted business hours, were imposed [25]. The Korean government regularly informed the public on the current level of ‘social distancing restriction’, which was different according to location. These procedures were enforced to restrict COVID-19 while also giving its citizens freedom when medically appropriate [26]. Despite these efforts, the government still received criticism from some citizens, stating that too many of their rights to freedom were prohibited due to restricted protests, religious gatherings, and even private gatherings when they consisted of more than a certain amount of people [18,26]. Even though reducing fatalities should be the main objective during a pandemic, privacy and the right to freedom should not be overlooked since public restrictions can lead to a lack of civil compliance and unrest [27,28]. Attempts to contain COVID-19 led to an economic depression in Korea; it had a 1.3% Gross Domestic Product (GDP) drop in 2022, with 476,000 people facing unemployment [29,30]. Retail owners and workers suffered heavily economically from the pandemic due to widespread fear of contracting the virus and restricted business hours.

The United States, compared to Korea, was less unified as a whole nation, carrying out more decentralized approaches to mitigate COVID-19. Due to the United States’ complex political system, where law and order differ by state autonomies, there were variations in regulations and guidelines between states and counties. Although the federal government provided general guidelines through the CDC, state and local governments had the decision to reject or accept guidelines [4]. In some states, the government enforced quarantine orders and restrictions on visiting non-essential places. On the contrary, other states resisted implementing statewide mandates, stating that it was not necessary and ineffective when preventing the spread. Variations in policy and enforcement in the United States were divided mostly by political beliefs across the nation [31].

**Figure 2.**
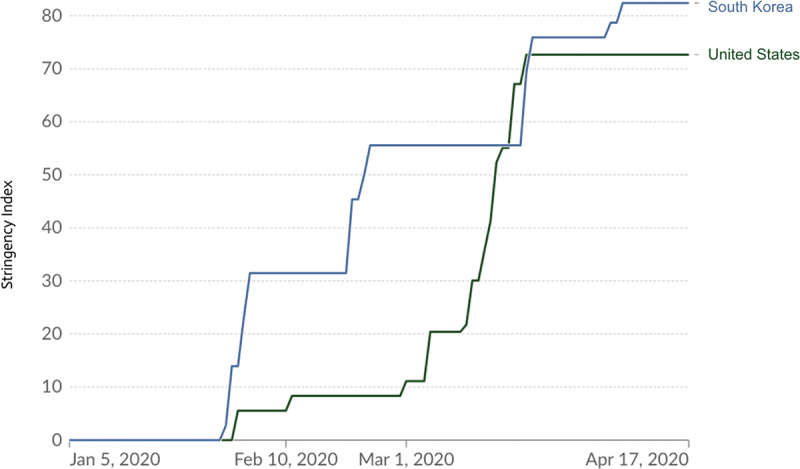
Stringency index of South Korea and the United States in early 2020 (reproduced with permission from OurWorldInData.Org).

The stringency index of a country is a composite measure of nine metrics to judge how strict a country’s response was, with the metrics being, ‘workplace closures; cancellation of public events; restrictions on public gatherings; closures of public transport; stay-at-home requirements; public information campaigns; restrictions on internal movements; and international travel controls,’ according to Our World in Data [32]. Since the Korean government extensively controlled its population using closures and lockdowns, especially during the start of the pandemic, it had a higher stringency index, as a higher index indicates stricter measures. Due to its dynamic four-tier system, the stringency index of Korea rose somewhat steadily over a longer period of time. On the other hand, the United States’ stringency index rose rapidly during March, going from below 10 to above 70 (Figure 2).

The rapid increase in the stringency index of the United States shows that closures, restrictions, and lockdowns were put into place immediately to control the spread of COVID-19. This could have led to social unrest and more confusion among citizens as they were unaware of which measures were in place in their local area. In addition, many people protested the strict measures the government was inflicting on them, refusing to comply, which could be due to a substandard information dissemination program the government created. The stringency index of Korea and the United States were at similar levels after peaking, proving that both countries did attempt to maintain strong control of their populations to further prevent the spread of COVID-19.

## Vaccination strategies and impact

The major turning point in the global struggle against the pandemic was the introduction of COVID-19 vaccines, which were extremely effective at limiting the virus from becoming life-threatening, helping widespread viruses become less fatal. Korea and the United States both had unique vaccination campaigns with different factors involved.

Originally, there was a delay in Korea’s vaccine rollout program since there were limited amounts of the vaccine received from countries that had successfully developed the vaccine earlier [33]. However, once Korea started its rollouts, the government strategized with its limited number of vaccines to primarily vaccinate specific high-risk populations, like healthcare workers and the elderly. The government, determined to make distribution accessible and organized, created a pre-vaccine registration system for citizens, enabling distribution to become accessible and organized. Korea launched a public education campaign confirming the safety of vaccines to combat skeptics and false information [33,34]. Shortly after, Korea underwent rapid vaccination progress, which played a role in the decline of mortality and morbidity due to the COVID-19 pandemic.

The rollout of vaccines in the United States took a unique path because of its decentralized healthcare system. The United States, thanks to the presence of pharmaceutical companies like Pfizer-BioNTech and Moderna, was one of the first few countries to develop a vaccine effectively preventing COVID-19. Therefore, the United States had access to vaccines much earlier than most other nations [35]. Nonetheless, the vaccination process was unorganized; there was a limited vaccination capacity, and the distribution and administration processes were delayed. While Korea exclusively approved certain hospitals to distribute vaccines, the United States also utilized health clinics and pharmacies [36]. Despite these issues, similarly to Korea, the United States vaccinated most of its population within a few months, reducing COVID-19 mortality.

The most prominent vaccines administered throughout Korea and the United States were Pfizer-BioNTech and Moderna, which both proved to be effective in preventing complications and fatalities due to COVID-19 [37,38].

BNT162b2 produced from Pfizer-BioNTech is 95.3% effective, while the mRNA-1273 vaccine produced from Moderna is 94.1% effective when blocking severe complications from COVID-19, according to the FDA [38,39].

**Figure 3.**
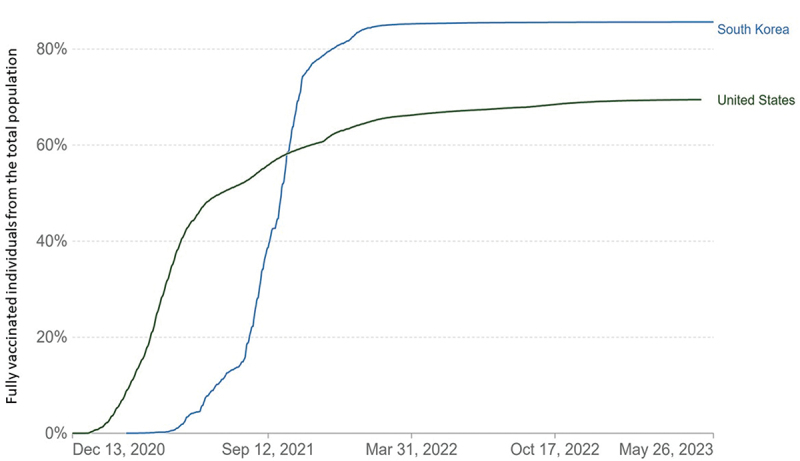
Percentage of the fully vaccinated individuals from total populations in the United States and South Korea between December 2020 to May 2023 (reproduced with permission from OurWorldInData.Org).

Even though the United States and Korea successfully distributed their vaccines, the vaccination rate between these two countries differed (Figure 3) – Korea demonstrated a higher overall vaccination rate, with 85.6% of the total population vaccinated at least twice (fully vaccinated) compared to the United States, with approximately only 70% of the population being fully vaccinated by December 2021 [40–42].

It is important to understand that vaccination rates in the United States varied significantly among states, with Massachusetts reporting a fully vaccinated rate of 82% among its population, while Alabama reported 52%, concluding that Massachusetts had a 30% higher rate of vaccination [41].

## Outcome analysis

The world, during a pandemic, should not only maximize its efforts to reduce cases but also reduce fatalities since it devastates society and the economy. Therefore, we evaluated Korea and the United States’s outcomes primarily using the mortality rate.

Before the end of 2022, when COVID-19 reached its deadliest point, the United States had 88.6 times more cases and 147.3 times more deaths than Korea, and since the United States possesses a 6.4 times larger population than Korea, the population-weighted difference could indicate that the United States had 13.8 times more cases and 23 times more deaths than Korea [40,43,44].

Korea, due to its aggressive response strategies, achieved a very low mortality rate. The worldwide average mortality rate was 0.09%; however, in Korea, the mortality rate was 0.07% [45]. In addition, thanks to widespread vaccination and robust patient care, the case fatality rate in Korea was 0.1%, about nine times lower than the global average.

**Figure 4.**
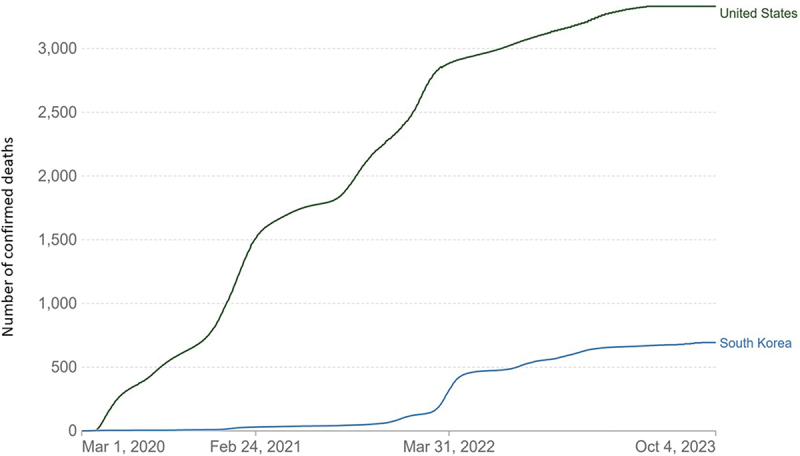
Cumulative deaths from COVID-19 per million people (reproduced with permission from OurWorldInData.Org).

By contrast, the United States faced a more severe outbreak with a higher case fatality rate and mortality (Figure 4); its fatality rate was 1.14%, more than 11 times higher than Korea, and its mortality rate was 0.34%, about five times higher than Korea [45,46].

## Results

We have analyzed the various COVID-19 response strategies that Korea and the United States implemented, from beneficial to ineffective decisions, and the unique challenges these countries faced (Table 1).

**Table 1.** Comparative analysis of responses to COVID-19 between South Korea and the USA.

Response to COVID-19	South Korea	United States of America
Previous experience	• 2015 **MERS** outbreak led to re-form its disease control mechanism. • Was equipped with refined response protocols, including accelerated testing and **contact trancing strategies** • Implemented **“Infectious Disease Control and Prevention Act”**	• Did not face a recent contagion akin to MERS before COVID-19 • Initial pandemic response systems tailored for **influenza-like outbreaks** • Faced challenges **adapting** to the novel coronavirus
Initial response	• Tested anyone who came **in contact** with infected people; had a **ten times higher test-to-confirmed-case ratio compared to US** • Established a **rigorous contact tracing** and isolating system using personal data prioritizing **safety over privacy**	• Had a **lower testing capacity** with a **lower test-to-confirmed-case ratio** • **Did not** implement a **stringent contact tracing system**, with **delays in expanding** it due to **privacy** and logistic concerns.
Information dissemination and public compliance	• Had an effective communication strategy that informed the public about the COVID-19 outbreak with **real-time alerts** • **Disclosed locations of infected people**; later received backlash for invasion for privacy • **90% of citizens** wore masks when going out	• Had a **lack of consistent communication** due to conflicting messages from government agencies • **75–80% of citizens** wore masks when going out; 10–15% lower than Korea’s compliance rate
Mitigation procedures	• Had **a four-tier social distancing system** to adjust the severity of their response accordingly to the virus’s spread • Encountered an **economic downfall** and a rise in unemployed people • Imposed **a fine** for non-compliance for the use of masks	• Employed a more **decentralized response**; implementing regulations were left to state and local governments • Variations of policy and enforcement were significantly based on **political beliefs**
Vaccination strategies and impact	• Had **a delay** when acquiring vaccines and starting a vaccine rollout • **High-risk populations** were prioritized for vaccination • Implemented a public education campaign regarding vaccine safety to combat **false rumors** • **85.6%** of the population was fully vaccinated until 2021	• Had **early access to vaccines** but disparate administration plans; different rollout strategies from state by state • **About 70%** of the population was fully vaccinated until 2021, **15.6% lower** than Korea’s percentage
Outcome	• **Positive**: very low case fatality rates; 11 times lower compared to the US, low mortality rates, widespread vaccination • **Negative**: privacy concern, economic depression	• **Positive**: consistent economy, faster recovery • **Negative**: higher case fatality and mortality rates, widespread vaccine misinformation

Subsequently, in this section we discussed the lessons to be learned and potential strategies to implement in order to be well-prepared for future crises.

### Effectiveness of policies in lowering the mortality rate

Korea, using its organized, centralized system, implemented extensive testing and contact tracing measures to limit the virus’ spread at the initial stage of the pandemic and acknowledged the nature of the virus, using appropriate tools to combat it. Korea had an accessible signup system for vaccination where a priority service was carried out for high-risk individuals, leading to brief and widespread vaccination across Korea, especially for the elderly. Furthermore, the government’s strategy to release information transparently and timely gained public adherence, lowering overall mortality rates during the pandemic.

By contrast, the United States’ responses were troublesome; providing inconsistent responses to the pandemic created delays when building an established prevention system. Initially, the virus rapidly spread as a consequence of the low testing capacity compared to its population, while the government made minimal efforts to speed up testing and contact tracing. The decentralized, state, and locally governed healthcare structure contributed to vast differences in mitigation procedures and vaccine distribution strategies, including the vaccination rate itself, resulting in unfavorable outcomes.

The policies of other East Asian countries that met our criteria above, Japan and China, were similar to those of Korea and had similar impacts. For example, Japan implemented the ‘Avoiding the Three C’s’ campaign, which was a nationwide campaign to refrain people from going to closed spaces, crowded places, and close-contact settings [47]. This was enforced by putting time-wise restrictions and lockdowns in those specific areas, eliminating spaces for COVID-19 to spread easily and curving the spread. The stringency of the measures in place fluctuated as the effects of COVID-19 worsened or became alleviated; however, unlike Korea, it did not have a specific numerical guideline in place to manage the stringency of the mitigation procedures. Similarly to Korea, China focused on specific case identification and large-scale surveillance during the initial stage of the pandemic, tracking down where each specific person with COVID-19 visited the past few days to put those spaces into lockdowns. A unique measure they implemented was that they installed infrared thermometers in major public spaces to track down anyone with a fever – these were also implemented in offices, and working areas, and even in the streets [48]. This could have contributed to a lower fatality rate in China as identifying COVID-19 before its symptoms worsen could lower the chance of severe consequences and resultant deaths. The data chart that summarizes the specific fatality, mortality, and vaccination rates of the various countries we studied is shown in Table 2.

**Table 2.** Data of COVID-19 Statistics of East Asian Countries and the United States.

	Worldwide	China	Japan	South Korea	United States
Total Cases	774,631,444	99,330,516	33,803,572	34,571,873	103,436,829
Total Fatalities	7,031,216	121,956	74,694	35,934	1,177,223
Fatality Rate	0.91%	0.12%	0.22%	0.10%	1.14%
Population	8,098,082,508	1,425,318,011	122,818,516	51,753,860	341,299,540
Mortality Rate	0.09%	0.01%	0.06%	0.07%	0.34%
Vaccination Rate*	67%	87%	82%	87%	70%

Note: *vaccination rate = complete primary series of vaccination.

### Unique challenges

Although Korea successfully contained the virus, its intensive contact tracing system posed privacy challenges. In addition, the public slowly started to denounce the government for restricting the freedom of assembly, association, and protest. During the vaccination process, the Korean government struggled to collect vaccines since they had to acquire them from countries that manufactured and distributed them, like the United States and the United Kingdom. By contrast, the United States struggled with disparities in healthcare, which caused different impacts on various communities, mainly due to socioeconomic factors. Many United States citizens were hesitant to receive a vaccination due to misinformation and political polarization regarding its efficacy.

### Lessons learned and future tactics

The most important lesson from Korea’s COVID-19 response is to carry out rigorous testing and contact tracing combined with timely information dissemination and vaccine distribution. From the United States’ strategies and outcomes, lessening health disparities, fighting misinformation and political divisiveness on health-related issues, and gaining public trust are the most important lessons to be learned and considered.

In the future, developing favorable pandemic response plans by investing in healthcare infrastructures and creating regulations and laws could be beneficial to preparing an effective pandemic response. Strictly enforcing regulations regarding the prevention of virus spread is another step toward limiting possible cases and enhancing the government’s authority over the spread of viruses. In addition, different health guidelines must be made for viruses with different characteristics, as COVID-19 was unique in having such a high fatality rate while being easily spreadable. Unlike an influenza-like virus with a lower fatality rate, to mitigate the effects of COVID-19 to prevent fatalities, the government should put in measures that are more focused on each individual case, such as specific contact contracting and immediate quarantine in spaces that are well-ventilated and have air purifiers with a medical-degree filter.

Although Korea is one of the countries with the lowest mortality rates during the COVID-19 pandemic and was praised worldwide for having a successful pandemic response, it was extensively intrusive in contact tracing and personal data collection. There is a need for discussion and resultant non-intrusive measures to be developed that can combat pandemics while respecting and balancing safety and privacy. For example, although specific personal details of people who were infected with COVID-19 were released in Korea, that is not necessary as it could breach the rights to privacy of citizens. The government should be able to track down specific people who were infected with COVID-19 and notify the ones in contact with them without releasing too much information, such as specific places they went.

The findings and lessons learned in this investigation not only provide a historical summary of the COVID-19 outbreak but also suggest a guide for future health crises. The management of pandemics is a complex endeavor that requires a coordinated and adaptable response strategy accompanied by public compliance. Although avoiding another severe health crisis would be favorable, viruses are unavoidable; using the lessons acquired from outbreaks, especially the COVID-19 pandemic, for the future is essential.

## Conclusion

Korea had a mortality rate nearly five times lower than the United States during the COVID-19 pandemic. It was discovered that major factors that could help a country achieve a lower mortality rate when fighting against a virus that has a high fatality rate are rapid response and testing, clear communication, and efficient vaccine distribution. Population control and more extensive contact tracing were common themes for East Asian countries that met our criteria, which had a lower overall mortality rate than the United States and the world. Future health guidelines that respect the rights of citizens’ freedom for viruses with higher fatality rates should be developed, as not finding the right balance could result in more fatalities or social unrest, which could lead to severe consequences.

## Limitations

It is essential to note that Korea took extensive measures and conducted many more tests per person than the United States. Some citizens who had no symptoms and would otherwise have not conducted a test were forced to test, revealing a positive result. Korea, as of now, has a higher case-to-population ratio than the United States since it has kept testing its citizens after the Omicron variant became widespread while the United States mostly stopped. This could affect the case fatality rate, a metric we used throughout this paper, since the Omicron variant has a much lower fatality rate than previous variants like the Delta variant.

In addition, readers should understand that although our indicators, which are initial response, information dissemination and public compliance, mitigation procedures, and vaccination strategies, were carefully selected to be compared in this research, there are many more factors that could have contributed to the difference in the case fatality rate and mortality rate. For instance, the testing procedure varied in these two countries; Korea most prominently used real-time PCR tests to diagnose COVID-19, while the United States utilized both real-time PCR tests and Rapid Ag Tests (RAT), which are proven to have a lower sensitivity rate, otherwise known as the true positivity rate.

This study compared countries from two distinct geographical areas; therefore, the findings may not be generalizable, as different countries across various regions have disparate socioeconomic circumstances and health infrastructures. However, the main lessons we discovered, as highlighted in our Conclusion, should be considered in many circumstances when creating public health guidelines and frameworks against future virus-led pandemics.
